# Multiple Opposing Constraints Govern Chromosome Interactions during Meiosis

**DOI:** 10.1371/journal.pgen.1003197

**Published:** 2013-01-17

**Authors:** Doris Y. Lui, Cori K. Cahoon, Sean M. Burgess

**Affiliations:** Department of Molecular and Cellular Biology, University of California Davis, Davis, California, United States of America; National Cancer Institute, United States of America

## Abstract

Homolog pairing and crossing over during meiosis I prophase is required for accurate chromosome segregation to form euploid gametes. The repair of Spo11-induced double-strand breaks (DSB) using a homologous chromosome template is a major driver of pairing in many species, including fungi, plants, and mammals. Inappropriate pairing and crossing over at ectopic loci can lead to chromosome rearrangements and aneuploidy. How (or if) inappropriate ectopic interactions are disrupted in favor of allelic interactions is not clear. Here we used an *in vivo* “collision” assay in budding yeast to test the contributions of cohesion and the organization and motion of chromosomes in the nucleus on promoting or antagonizing interactions between allelic and ectopic loci at interstitial chromosome sites. We found that deletion of the cohesin subunit Rec8, but not other chromosome axis proteins (e.g. Red1, Hop1, or Mek1), caused an increase in homolog-nonspecific chromosome interaction, even in the absence of Spo11. This effect was partially suppressed by expression of the mitotic cohesin paralog Scc1/Mdc1, implicating Rec8's role in cohesion rather than axis integrity in preventing nonspecific chromosome interactions. Disruption of telomere-led motion by treating cells with the actin polymerization inhibitor Latrunculin B (Lat B) elevated nonspecific collisions in *rec8*Δ *spo11*Δ. Next, using a visual homolog-pairing assay, we found that the delay in homolog pairing in mutants defective for telomere-led chromosome motion (*ndj1*Δ or *csm4*Δ) is enhanced in Lat B–treated cells, implicating actin in more than one process promoting homolog juxtaposition. We suggest that multiple, independent contributions of actin, cohesin, and telomere function are integrated to promote stable homolog-specific interactions and to destabilize weak nonspecific interactions by modulating the elastic spring-like properties of chromosomes.

## Author Summary

Meiosis is the key stage of gametogenesis, when the diploid genome complement is reduced by one half to form haploid gametes for sexual reproduction. Accurate chromosome segregation requires that homologous chromosomes pair, recombine by crossing over, and segregate from one another during the first meiotic division. Missegregation of homologs leads to the formation of aneuploid gametes, while erroneous crossing over between ectopic chromosomal loci can lead to chromosomal rearrangements such as translocations and deletions. We found that nonspecific interactions between interstitial chromosomal sites can be enabled or prevented through multiple, independent mechanisms during meiosis in budding yeast. These include organization of chromosomes in the nucleus, integrity of the chromosome axis structure, and actin-led chromosome movement. Acting together, these processes can reinforce strong chromosome interactions that promote pairing, while acting in opposition they can eliminate weak nonspecific interactions. These data provide an integrated view of how homologous chromosome pairing is achieved.

## Introduction

Meiosis is a specialized cell division program that generates haploid gametes from diploid parental cells. A hallmark of the meiosis I division is the reductional segregation of homologous chromosomes while the meiosis II division segregates sister chromatids. The reductional division requires crossing over between homologous chromosomes in combination with sister chromatid cohesion [1], [2]. Errors preventing normal chromosome segregation are a major cause of birth defects and miscarriage [3].

Crossing over is the outcome of reciprocal exchange of chromosome segments of homologous nonsister chromatids. Typically exchange occurs at allelic positions on homologous chromosomes but can also occur erroneously between ectopic regions of homology located on nonhomologous chromosomes, resulting in deletions, insertions and/or translocations [4]–[7]. Over the past decade, major inroads have been made in understanding mechanisms that promote pairing between homologous chromosomes but little is known about the mechanisms that prevent nonallelic interactions [8]. Several lines of evidence point to the sequestration of repeated elements to “silenced” regions near the nuclear periphery [7], [9]–[11] or through engagement with allelic DNA sequences by homologous recombination [12], [13].

The relationship between events that initiate crossing over and mechanisms that promote the side-by-side alignment of homologs varies among species [14]. In a majority of model organisms studied, including mouse, plants and fungi, the repair of Spo11-induced double-strand breaks (DSBs) using the homologous chromosome as a repair template is a major driver of pairing [15]–[23]. By contrast, in *Caenorhabditis elegans* and *Drosophila* females where recombination is still required for proper disjunction, homologs can pair even in the absence of meiotic-induced DSBs [24]–[26]. In *C. elegans*, pairing is initiated at pairing centers found at one end of each chromosome [27]. In *Drosophila* females, achiasmate chromosomes can pair via regions of heterochromatin [24], [25], [28]. In *Drosophila*, and to a lesser extent in budding yeast, an alternative mechanism to segregate achiasmate chromosomes exists that relies on homolog nonspecific interactions between centromere sequences [29]–[31].

While full levels of pairing in budding yeast *Saccharomyces cerevisiae* requires the formation and repair of DSBs there is also evidence for DSB-independent pairing both in vegetatively dividing cells and during meiosis in (e.g. in a *spo11*Δ mutant) [32]–[36]. The configuration of chromosomes with respect to centromere and/or telomere clustering and chromosome territories contributes in part to associations between homologous chromosomes at these regions [8], [13], [37], [38]. Centromere coupling is an early event during meiotic prophase that involves pairwise associations of centromeres independent of homology [29], [35]. Examples abound from a wide variety of species for somatic homolog pairing in higher eukaryotes with direct influence on gene expression or DNA repair [7], [39].

The structural core of the meiotic chromosome axis in budding yeast comprises a conserved group of proteins, including Rec8, a meiosis-specific α-kleisin subunit of cohesin as well as Red1, Hop1 and Mek1 [40]–[46]. Inactivation of any of these proteins compromises interhomolog bias and homolog pairing [15], [18], [21], [47]–[53]. Deletion of Rec8 also impacts several events of meiotic prophase not associated with sister chromatid cohesion, including region-specific distribution of Spo11 and DSB formation along chromosomes, meiotic S-phase timing, centromere coupling, synapsis, homolog pairing, transcription, and progression through meiotic prophase [40], [44], [49], [51], [54]–[58]. Rec8 also plays an important role in chromosome segregation at meiosis I by preventing premature sister chromatid separation prior to anaphase I [40].

In addition to the biogenesis of specialized chromatin architecture, meiotic chromosomes of nearly all species assume a polarized, nonrandom configuration in the nucleus, often with telomeres clustered toward one side of the nucleus [59]. This configuration is associated with vigorous telomere-led movement driven by cytoskeleton structures (either actin or microtubules depending on the species) outside the nucleus through protein bridges that span the inner and outer nuclear membranes and attach to telomeres [60], [61]. In budding yeast, the velocity of telomere-led movement is greatest during late zygotene to pachytene stages when homologs are already paired, however, slower chromosome movement can be observed prior to zygotene during the pairing stage [62]–[65]. Chromosome organization and motion appear to be coupled to events associated with pairing and recombination since nearly every mutation affecting one or both of these aspects also exhibits slow turnover of recombination intermediates and delayed pairing.

Meiotic chromosomes are mechanically linked to the cytoskeleton through the intact nucleus by a conserved SUN-KASH protein bridge [66], [67]. Ndj1 is a fungal-specific telomere-associated protein that promotes telomere/NE associations [63], [68]–[70]. Ndj1 interacts with the conserved SUN-protein Mps3 that spans the inner nuclear envelope [71]. Mps3 interacts with Csm4, a putative KASH protein with a single trans-membrane tail domain bridging the outer nuclear envelope; Csm4 is required for telomeres to coalesce into the bouquet configuration and undergo Ndj1-dependent motion [63], [64], [72], [73].

This work focuses on how the structure and organization of chromosomes in the nucleus impacts interactions between allelic and ectopic interstitial chromosomal loci. Here we carried out extensive epistasis analysis using deletion mutations in genes known to be involved in each of the functions described above to define the co-dependent or independent pathways leading to close-stable homolog juxtaposition (CSHJ). We applied an *in vivo* assay (Cre/*loxP*) that measures the relative spatial proximity/or accessibility of pairs of chromosomal loci [32]. Maximal levels of site-specific recombination between homologous chromosomes indicated close, stable homolog juxtaposition of the assayed interstitial loci [21]. Through the analysis of mutants defective for various processes related to meiotic recombination we found that early steps of homologous recombination, including strand invasion and single end invasion are major determinants of CSHJ, while synapsis plays a relatively minor role [21], [74], [75].

## Results

### The experimental system

**Figure 1:**
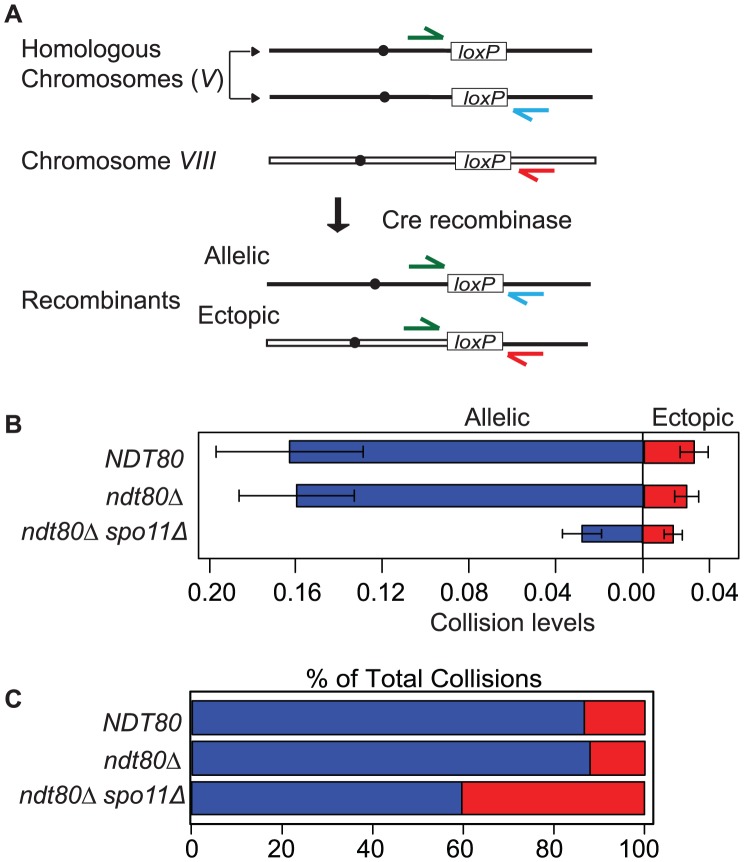
Inter-chromosomal collision assay. A. The chromosomal location of *lox*P sites for the collision assay described in the text. The primer configurations for detection of recombinants by qPCR are diagrammed. B. Comparison of average allelic and ectopic collision levels in *NTD80* versus *ndt80*Δ and*ndt80*Δ *spo11*Δ. Collisions are the measured level of recombinants per meiosis (i.e. 4 chromatids). The variance for allelic collision levels was somewhat higher than for ectopic. This may be due to differences in primer sequence and primer pair concentrations optimized for the respective qPCR reaction conditions; the template DNA isolated from each individual time course for both cases is the same. C. Percent of allelic collisions (blue) and ectopic collisions (red) among total collisions measured (allelic and ectopic).

To probe the spatial proximity and/or accessibility of pairs of interstitial chromosomal loci *in vivo*, we measured the frequency of Cre-catalyzed recombination between pairs of *loxP* sites located at allelic and ectopic chromosomal loci per meiosis. These sites were integrated at positions equidistant from the centromere and the adjacent telomere on the long arms of two average sized chromosomes (*V* and *VIII*; see Experimental Procedures for more details). Previously, we measured site-specific recombination events by selecting for prototrophs resulting from the coupling of a promoter region to a selectable reporter gene; prototrophs were recovered from synchronized meiotic cells plated on selective media by “return to growth” (RTG) at various time points after the initiation of meiosis (by transfer to sporulation medium; SPM [21]). In this study we measured recombinant DNA products by quantitative PCR using chromosome-specific primers flanking each *loxP* site (Figure 1A). One key advantage of using qPCR is that Cre/*lox*P recombination can be assessed in strains that do not survive RTG and processing samples is more efficient. Template DNA for PCR was isolated from cells collected 10 hours after the initiation of meiosis. The recombination events are normalized for each sample by dividing by the copy number of a control locus that does not undergo Cre/*loxP* recombination (*ACT1*). By multiplying the normalized value by the total number of chromatids per chromosome (four chromatids for most of the strains analyzed in this study), we generate the number of Cre-mediated recombination events per meiosis. The output of this assay, for both the RTG and qPCR method, is the frequency of Cre/*lox*P recombination events per meiosis, which we will refer to here as “collisions” (Figure 1A).

Strains used in this analysis carry an *ndt80*Δ mutation to prevent pachytene exit; blocking this late prophase step provides a control for differences in division timing and/or arrest exhibited by various meiotic mutants [76]. Prolonged arrest at pachytene does not affect the output of the assay since *NDT80* and *ndt80*Δ strains gave similar levels of both allelic and ectopic collisions (Figure 1B).

It is important to note that collision levels represent the cumulative events that occur from the time of bulk DNA replication, when Cre recombinase is induced by galactose addition, until a fixed arrest point prior to exit from pachytene. Galactose induction increased allelic and ectopic collision levels 100- and 350-fold compared to those in untreated cells, respectively (Table S1); thus, the dynamic range spans two orders of magnitude while background level of events is negligible.

Collision levels measured using the qPCR and RTG methods were in agreement: Allelic collision levels were 0.13±0.03 and 0.14±0.03 recombinants/4 chromatids for qPCR and RTG values, respectively; ectopic collision levels were 0.018±0.004 and 0.015±0.004, respectively (Figure S1). From previous studies, we have inferred that the elevated level of allelic versus ectopic collisions is due to homology-dependent interhomolog interactions in sequences outside the reporter locus [21], [74], [75], [77]. A number of mutants defective for meiotic recombination including *spo11*Δ, *sae2*Δ, *rad51*Δ, and *rad52*Δ exhibited reduced allelic collision levels compared to wild type when measured by qPCR and RTG assays (Figure S1). Additional mutants defective for biochemical aspects of recombination including *zip3*Δ, *rdh54*Δ, and *sgs1-mn* (a meiotic null allele of *SGS1*) were analyzed in the course of this study but not discussed here (Figures S2, S3 and Table S2; [78]–[83]).

### Rec8 promotes allelic collisions independent of its role in sister chromatid cohesion

Rec8 plays dual roles during meiotic prophase; the first is to mediate sister chromatin cohesion and the second as a structural component of the chromosome axis that regulates the position and outcomes of homologous recombination (Introduction). To explore if one or both of these functions influence the proximity and/or accessibility of interstitial chromosomal loci, we measured allelic and ectopic collision levels in a strain expressing the mitotic cohesin *SCC1/MCD1* in place of *REC8* (*pREC8-SCC1*) in the *rec8*Δ mutant. In *pREC8-SCC1*, sister chromatid cohesion is maintained while meiosis-specific functions, including chromosome axis and SC assembly are absent [49], [56], [84]. In *rec8*Δ mutants, both aspects of Rec8 function are absent.

**Figure 2:**
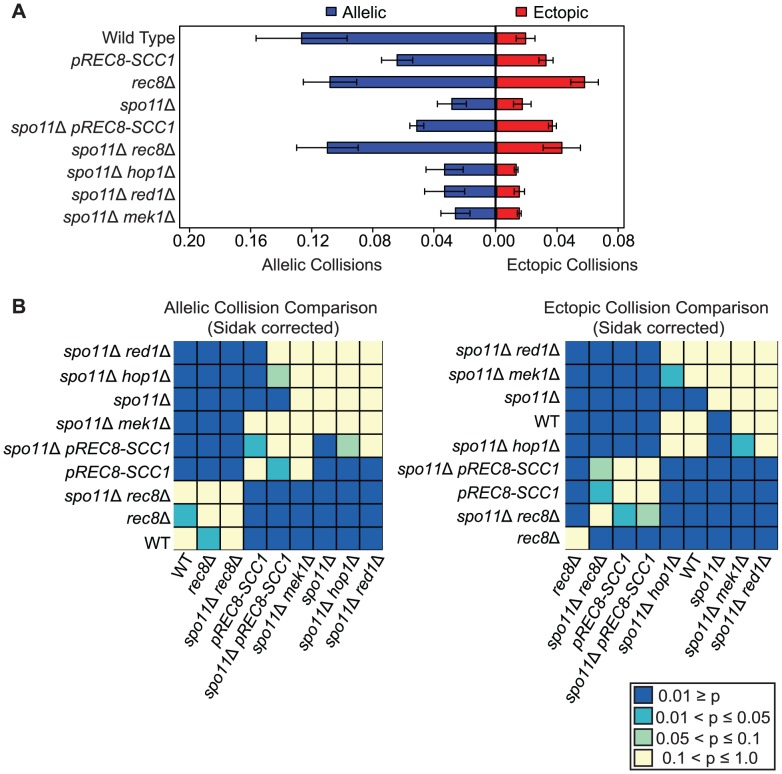
Rec8 promotes allelic collisions independent of its role in sister chromatid cohesion and prevents nonspecific collisions. A. Analysis of allelic and ectopic collision levels as described in Figure 1. B. Heatmap of Sidak adjusted *P*-values comparing collision levels between all allelic collision levels (left) and all ectopic collision levels (right).

We found that *pREC8-SCC1* reduced the level of allelic collisions 2-fold compared to wild type (0.064±0.009 versus 0.127±0.031, *P*<0.00001; Figure 2) implicating a role for Rec8 in promoting allelic chromosome interactions independent of its role in sister chromatid cohesion. Brar *et al.* reached a similar conclusion by analyzing pairing in individual cells using GFP-tagged chromosomes in this mutant background [49]. To our surprise, we found that the level of allelic collisions and ectopic collisions in *rec8*Δ (i.e. the absence of cohesion) was greater than in the *pREC8-SCC1* (*P* = 6.04e^−11^; Figure 2). In addition, the level of ectopic collisions was elevated 3-fold in the *rec8*Δ mutant compared to wild type (0.058±0.009 versus 0.019±0.006, *P* = 1.04e^−13^) and *pREC8-SCC1* (0.058±0.009 versus 0.032±0.004, *P* = 3.43e^−9^). Together these results suggest that Rec8 promotes interhomolog interactions and suppresses ectopic interactions, and perhaps nonspecific allelic interactions (below). These effects may be region-specific since the regions including *FLO8 (V)* and *NDT80 (VIII)* are enriched for Rec8 binding and exhibit decreased levels of DSBs in *rec8*Δ mutant cells compared to wild type [85]–[87].

### Nonspecific collisions are elevated in both *rec8Δ* and *pREC8-SCC1* mutants

We reasoned that the relatively modest reduction in the allelic collision level conferred by *rec8*Δ compared to *pREC8-SCC1* might be due to the inclusion of a significant fraction of “nonspecific” interactions (i.e. those occurring in the absence of homology-dependent interactions). To test this, we measured the level of collisions in a *spo11*Δ mutant background since homologous recombination is a major driver of allelic collisions in wild type strains [75]. We found that the level of allelic collisions in *spo11*Δ *rec8*Δ was 3.9-fold greater than in the *spo11*Δ single mutant (0.110±0.020 versus 0.028±0.009, *P* = 1.49e^−11^ respectively; Figure 2). Allelic collisions in *spo11*Δ *pREC8-SCC1* were also greater than *spo11*Δ (2-fold; 0.055±0.011 versus 0.028±0.009, *P* = 1.72e^−5^ respectively; Figure 2), but to a lesser extent than *spo11*Δ *rec8*Δ. Likewise, ectopic collision levels were elevated in *spo11*Δ *rec8*Δ and *spo11*Δ *pREC8-SCC1* compared to *spo11*Δ (*P* = 0.0003 and *P* = 7.88e^−9^, respectively; Figure 2). This trend was also observed, but to a lesser extent, in a *spo11*Δ *rec8*Δ *cdc6-mn* where cells progress through meiosis without fully duplicating the parental chromosomes (Figures S2, S3; [88]). These results suggest that the loss of Rec8 cohesion function (but not loss of sister cohesion *per se*) leads to increased interactions between chromosomal loci independent of DSB formation.

### Nonspecific collisions are not elevated in the absence of Red1, Hop1, or Mek1

We next tested if other components of the meiotic chromosome axis (Red1, Mek1 and Hop1) limit nonspecific collisions similar to Rec8. Unlike the case for *spo11*Δ *rec8*Δ, however, we found that the levels of allelic and ectopic collisions in *spo11*Δ *red1*Δ, *spo11*Δ *mek1*Δ and *spo11*Δ *hop1*Δ mutants were indistinguishable from *spo11*Δ, with the exception of *spo11*Δ *hop1*Δ in which ectopic collision levels were slightly reduced (*P* = 0.005; Figure 2). These results further indicate that the effect of the *rec8*Δ on increasing chromosome interactions is due the absence of cohesin and not by disrupting the core axis structure.

### The increase in nonspecific collisions in *rec8*Δ is not due to the persistent bouquet

**Figure 3:**
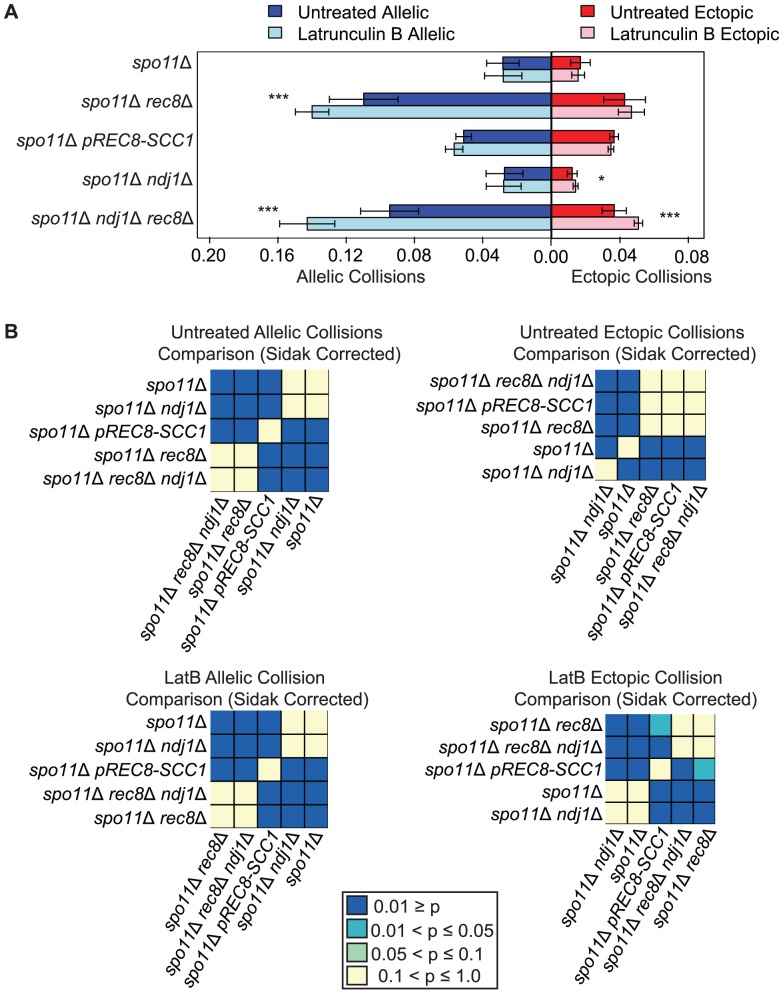
Elevated levels of nonspecific collisions in *rec8*Δ mutants do not require recombination initiation. A. Analysis of collisions in *spo11*Δ, *spo11*Δ *rec8*Δ, *spo11*Δ *prec8-SCC1, spo11*Δ *ndj1*Δ, and *spo11*Δ *ndj1Δ rec8*Δ mutants with Lat B treatment. Allelic (blue) and ectopic (red) collision levels in untreated cultures (dark bars) and Lat B treatment (light bars). Asterisks denote significant differences as follows: (*), *P*-values between 0.05 and 0.01; (**), *P*-values between 0.01 and 0.001; (***), *P*-values <0.001 by a two-tailed Student's t-test. B. Heatmap of Sidak adjusted *P*-values from Student's t-test comparing collision levels between relevant mutants in untreated and Lat B treated cells.

We next tested the possibility that the relatively high level of nonspecific chromosome interactions in *spo11*Δ *rec8*Δ is due to the juxtaposition of loci located at similar chromosomal “latitudes” in the bouquet configuration since the bouquet persists in this mutant background [53]. We reasoned that disrupting the bouquet might reverse the increased levels of nonspecific interactions conferred by *rec8*Δ. To test this we deleted *NDJ1*, encoding a telomere-associated protein that promotes attachment of chromosome ends to the nuclear envelope, and assayed collisions under this condition where the bouquet is absent [70], [73], [89]. We found that the levels of both allelic and ectopic collisions were similar in *spo11*Δ *rec8*Δ *ndj1*Δ and *spo11*Δ *rec8*Δ (*P* = 0.3 and *P* = 0.8 respectively) suggesting that a persistent bouquet is not responsible for increased collision levels in the *rec8*Δ background (Figure 3). Interestingly a significant reduction in ectopic collisions was found in the control strain *spo11*Δ *ndj1*Δ compared to *spo11*Δ (0.012±0.004 and 0.017±0.006 respectively, *P* = 0.003; Figure 3). This finding was further explored as described below.

### Addition of the actin polymerization inhibitor Lat B further increases nonspecific collisions in *spo11*Δ *rec8*Δ

We next speculated that the high levels of nonspecific interactions observed in *spo11*Δ *rec8*Δ might be driven by actin-mediated motion. To test this, we added the actin polymerization inhibitor Latrunculin B (Lat B) to cell synchronized meiotic cultures and asked if it reduced the levels of allelic and ectopic collisions [90]. We were surprised to find that the level of allelic collisions was instead increased in *spo11*Δ *rec8*Δ cells treated with Lat B compared to untreated cells (0.140±0.010 vs. 0.110±0.020; *P* = 1.9e^−5^). This trend was also observed for ectopic loci (0.047±0.008 vs. 0.043±0.012, albeit above the threshold of significance; Figure 3). This outcome suggests that actin can antagonize nonspecific interactions. There was no measurable effect of Lat B on the *spo11*Δ single mutant. Moreover, Lat B treatment did not significantly affect the level of allelic collisions in the *pREC8-SCC1 spo11*Δ mutant where sister chromatid cohesion exists (Figure 3). These results suggest that the cohesin function of Rec8 acts in opposition to an actin-based mechanism to suppress nonspecific chromosome interactions.

If the elevated level of nonspecific interactions in *spo11*Δ *rec8*Δ were due to Ndj1-dependent, telomere-led motion we would expect that the addition of Lat B to *spo11*Δ *rec8*Δ *ndj1*Δ would have no effect. Instead, we found that Lat B elevated both allelic collisions (0.143±0.016 versus 0.095±0.017; *P* = 0.0001; Figure 3) and ectopic collisions (0.051±0.002 versus 0.037±0.007; *P* = 0.0002; Figure 3) by approximately 50% even in the absence of Ndj1. Without the combined constraints of Ndj1-dependent attachment of chromosomes to the nuclear envelope, Rec8-mediated cohesion and an unknown feature of actin (i.e. Lat B treated *spo11*Δ *rec8*Δ *ndj1*Δ), the total level of collisions (i.e. the sum of allelic and ectopic events) is 4.3 fold greater than when these constraints are intact (i.e. in a *spo11*Δ single mutant). Thus, even in the absence of DSBs, multiple independent processes appear to impose chromosome order within the 3D space of the nucleus.

### Addition of Lat B elevates allelic collisions in *csm4*Δ and *ndj1*Δ mutants

**Figure 4:**
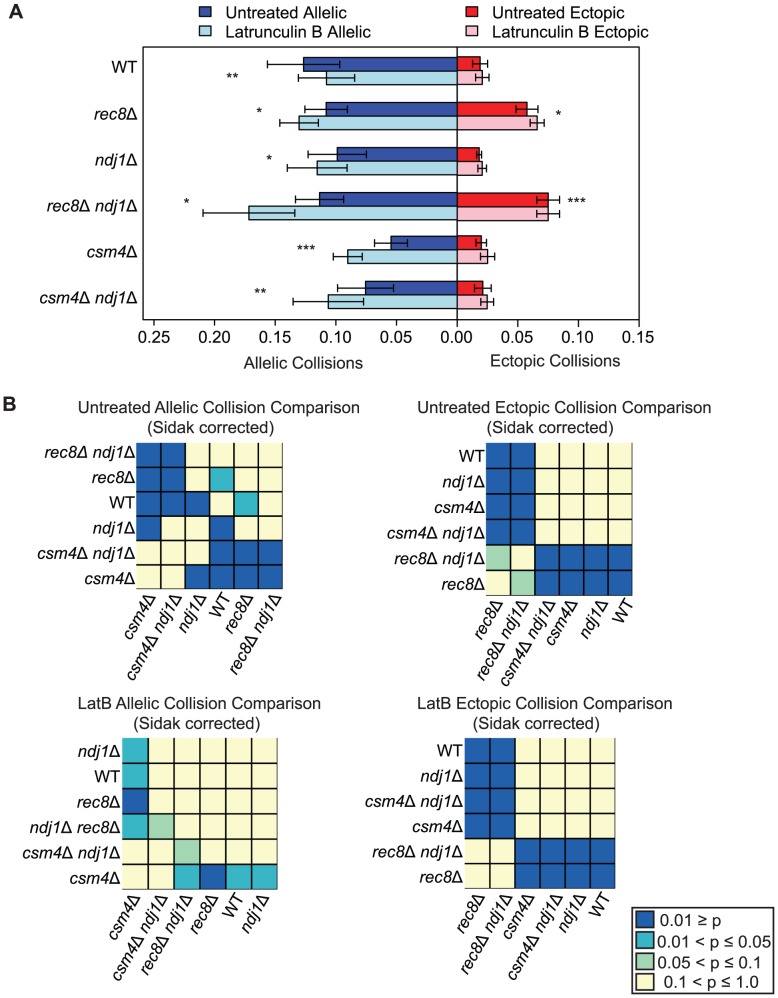
Elevated levels of nonspecific collisions in *rec8*Δ do not require Ndj1-dependent telomere attachments to the NE. A. Analysis of allelic and ectopic collision levels in *rec8Δ*, *ndj1*Δ, *ndj1*Δ *rec8*Δ, *csm4*Δ, and *ndj1*Δ *csm4*Δ mutants with Lat B treatment. Graph parameters are as described as in Figure 3. B. Heatmap of Sidak adjusted *P*-values from Student's t-test comparing collision levels between relevant mutants in untreated and Lat B treated cells.

The addition of Lat B to wild-type cells does not affect overall levels of homolog pairing assayed using FISH, however, it delays the kinetics of pairing compared to untreated cells [53]. When Lat B was added to wild type (e.g. *SPO11*) cells the level of allelic collisions was modestly reduced to 84% of untreated cells (*P* = 0.003; Figure 4) the level of ectopic collisions remained unchanged (*P* = 0.2; Figure 4), suggesting that actin can play a positive role in promoting recombination-mediated allelic interactions in addition to antagonizing nonspecific interactions (above). This result is not surprising since the levels of crossover-bound recombination intermediates (Single End Invasions (SEIs) and double Holliday Junctions (dHJs)) are not dramatically reduced in Lat B-treated cells [62].

By contrast, we found that the addition of Lat B increased levels of allelic collisions in *rec8*Δ, *ndj1*Δ, *csm4*Δ, *rec8*Δ *ndj1*Δ and *ndj1*Δ *csm4*Δ compared to untreated cells (Figure 4). These results suggest that in the absence of telomere-led movement or dynamic nuclear deformations, a Lat B sensitive process can negatively influence allelic chromosome interactions. Interestingly, only *rec8*Δ and *rec8*Δ *ndj1*Δ mutants significantly increase ectopic collision levels and Lat B further increases these collisions (*P* = 0.02; *P* = 0.0004 respectively, Figure 4). These results are consistent with the findings shown above (Figure 3) suggesting a role for Rec8 in constraining nonspecific chromosome interactions. Moreover, these data implicate actin in a nuclear process independent of Ndj1-dependent telomere-led motion.

### Lat B disrupts pairing of GFP-tagged chromosomal loci in wild type, *ndj1*Δ, and *csm4*Δ

**Figure 5:**
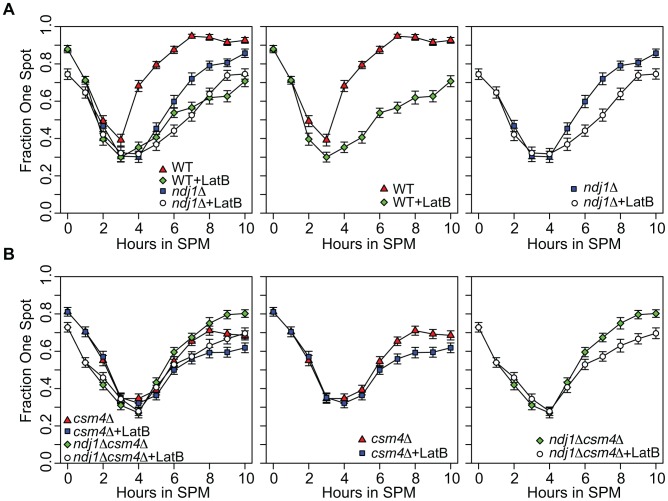
Homolog pairing kinetics in wild-type, *ndj1*Δ, *csm4*Δ, and *ndj1*Δ *csm4*Δ cells with and without Lat B treatment. Kinetics of pairing in cells with *tetO* arrays integrated at *URA3*, located 35 kb away from the centromere on chromosome V, and expressing tetR-GFP fusion protein. Homologs are considered paired if only one GFP can be visualized in the cells (n = 200) from each time point. Error bars represent the standard error of the percentage of cells paired for independent cultures for each mutant (n = 200). All strains carry the *ndt80*Δ mutation. A. Analysis of WT (center panel and left panel) and *ndj1Δ* (right panel and left panel) pairing kinetics in the presence or absence of Lat B. B. Analysis of *csm4*Δ (center panel and left panel) and *ndj1*Δ *csm4*Δ (right panel and left panel) pairing kinetics in the presence or absence of Lat B.

We next used an independent visual assay to measure the effect of Lat B on chromosome interactions using strains expressing a TetR-GFP fusion protein that binds integrated *tet*O arrays at the *URA3* locus on homologous chromosomes [91]–[93]. As was also observed by Brar *et al.*, for wild type, the two loci colocalize forming one focus prior to transfer of cells to SPM. As cells enter meiosis, colocalization is progressively reduced up until about t = 3 hours (Figure 5A; [49]). While untreated cells reach maximum levels of pairing by t = 7 hours (∼90% have one GFP spot; Figure 5A), pairing in Lat B treated cells was delayed and only ∼55% had one spot at this time point. Thus, the effect of Lat B on pairing using this visual assay was more severe than using the collision assay.

Pairing kinetics in the untreated *ndj1*Δ mutant were delayed compared to wild type, similar to observations made using FISH [70]. This delay was even greater in Lat B-treated cells. Indeed, the kinetics of pairing in both wild type and *ndj1*Δ cells treated with Lat B were similar (Figure 5A). These results suggest 1) Ndj1 does not play a role in promoting pairing other than through its actin-related function and 2) that actin promotes pairing of allelic sites, in part, through a process that acts independently of Ndj1. We did not observe a recapitulation of the collision phenotype where Lat B stimulates allelic collisions. Since the effect of Lat B appears to have an opposite effect in the GFP assay compared to the collision assay in the *ndj1*Δ, it is apparent that they measure different aspects of meiotic chromosome dynamics.

In the absence of Ndj1, there is a considerable degree of Lat B-sensitive dynamic nuclear deformation [94]. Since dynamic nuclear deformations require the putative KASH protein, Csm4 [94], we reasoned that addition of Lat B to *csm4*Δ or *csm4*Δ *ndj1*Δ would have little or no effect on the kinetics or absolute levels of pairing. This turned out not to be the case, however, since addition of Lat B reduced and/or delayed pairing levels in both mutants (Figure 5B). Together, these results suggest that an actin-mediated process and/or structure positively influences homolog pairing independent of Ndj1-dependent telomere-led motion or dynamic nuclear deformations.

### Ndj1 and Zip1 together promote interactions between ectopic chromosomal loci in the absence of DSBs

**Figure 6:**
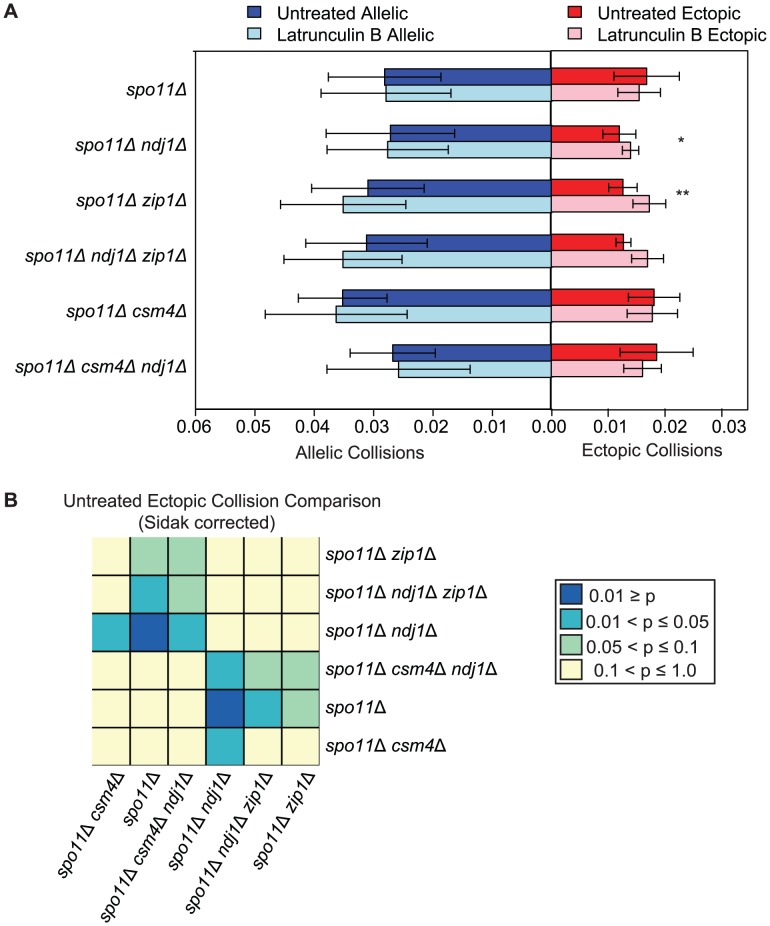
DSB–independent ectopic collision levels in mutants with defects in centromere coupling and bouquet formation. A. Analysis of collisions in *spo11Δ, spo11Δ ndj1Δ, spo11Δ zip1Δ, spo11Δ ndj1Δ zip1Δ, spo11Δ csm4Δ* and *spo11Δ csm4Δ ndj1Δ* mutants with Lat B treatment. Graph parameters are as described in Figure 3. B. Heatmap of Sidak adjusted *P*-values from Student's t-test comparing collision levels between relevant mutants in untreated and Lat B treated cells.

During early meiotic prophase, chromosomes in yeast are loosely organized by centromere coupling and attachment of telomeres to the nuclear envelope. Since this configuration does not require DSB formation, one widely held notion is that this arrangement of chromosomes may precede and set the stage for DSB-mediated pairing, similar to DSB independent pairing of heterochromatin in *Drosophila* or pairing centers in *C. elegans* [14], [35], . We reasoned that collision levels might reflect this organization and that disruption of telomere attachment (e.g. *ndj1*Δ) and/or centromere coupling (e.g. *zip1*Δ) would reduce ectopic collision levels. To overcome the strong effects of homologous recombination, we carried out the analysis in a *spo11*Δ mutant background. Interestingly, ectopic collision levels were reduced in both the *spo11*Δ *ndj1*Δ and *spo11*Δ *zip1*Δ double mutants to 73% (*P* = 0.003) and 75% (*P* = 0.03) of *spo11*Δ levels, respectively (Figure 6). Notably, these are the only mutant situations for which we have observed a reduction in ectopic collisions either in the presence or absence of DSBs for dozens of analyzed mutant strains (Figure S2; [21], [74], [75]).

If the observed decrease in ectopic collision levels was due to the independent contributions of Ndj1 and Zip1, we expected that the *spo11*Δ *zip1*Δ *ndj1*Δ triple mutant would give even lower collision levels compared to either *spo11*Δ *ndj1*Δ or *spo11*Δ *zip1*Δ double mutants. Interestingly, however, ectopic collision levels in double and triple-mutant strains were indistinguishable (*P*>0.99 for both cases; Figure 6). These collision levels (∼0.013) are two orders of magnitude above the lower limit of detection using this assay (i.e. *P*<0.00005; see Table S1), so a decrease should be detectable, in principle. These data suggest that Ndj1 and Zip1 participate in a single process that facilitates a subset of interactions between ectopic interstitial loci. For example, the simultaneous occurrence of telomere/NE tethering and centromere coupling could promote alignment of similar-sized chromosome arms, irrespective of homology. To further test this model, collisions at additional chromosomal sites must also be analyzed.

### Evidence for weak forces destabilized by actin

To our surprise, Lat B added to *spo11*Δ *ndj1*Δ and *spo11*Δ *zip1*Δ double mutants restored ectopic collision levels to the same as *spo11*Δ (1.22-fold, *P* = 0.04 and 1.37-fold, *P* = 0.003, respectively). This was also true when Lat B was added to the *spo11*Δ *zip1*Δ *ndj1* triple mutant (1.33-fold, *P* = 0.01). By contrast, ectopic collisions in *spo11*Δ were virtually the same in untreated and treated cells (0.0173±0.006 *vs.* 0.0154±0.004; *P* = 0.11, respectively; Figure 6 light blue bars). Thus, there appear to be weak stabilizing forces between ectopic sites maintained in the absence of Ndj1 and/or Zip1 that are sensitive to disruption by an actin mediated process or event.

We next tested if dynamic nuclear deformations are responsible for disrupting these weak ectopic interactions by introducing the *csm4*Δ mutation into these strains. In this case we would expect that the *csm4*Δ mutation would prevent destabilization and thus phenocopy the effect of Lat B treatment. This indeed turned out to be the case since ectopic collisions in *spo11*Δ *ndj1*Δ were increased to *spo11*Δ levels in the absence of Csm4 (*P* = 0.94; Figure 6). Importantly, addition of Lat B to *spo11*Δ *csm4*Δ and the *spo11*Δ *ndj1*Δ *csm4*Δ triple mutant did not increase ectopic collisions indicating that Csm4 acts in the same pathway as an actin-mediated process (perhaps by mediating dynamic nuclear deformations) that destabilizes weak interactions between ectopic loci.

## Discussion

Our goal was to understand how the nonrandom organization of chromosomes in the nucleus, including the contributions of actin-driven motion, promotes stable homolog juxtaposition and/or limits nonspecific interactions during meiosis prophase I. We used a quantitative “collision” assay to measure the relative proximity and/or accessibility of allelic and ectopic pairs of interstitial chromosomal loci in various mutant strains of yeast defective for aspects of meiotic chromosome dynamics. We expanded the scope of our previous studies demonstrating that the repair of meiosis-induced DSBs plays a prominent role in achieving close, stable homolog juxtaposition [21], [74], [75]. We found evidence that supports roles for Ndj1/Csm4 and actin-driven motion in homolog pairing. We found that a combined function of Ndj1/Zip1 facilitates nonspecific chromosome interactions, perhaps by aligning similarly sized chromosomes engaged simultaneously in centromere coupling and telomere/NE attachment. Finally, we uncovered several independent mechanisms that antagonize nonspecific chromosome interactions, including a cohesion function of Rec8 and more than one process involving actin. We propose that close, stable homolog juxtaposition in yeast is achieved through a balance of forces that promote strong homolog specific interactions and destabilize (or prevent) weak nonspecific interactions. The discussion below describes how these multiple opposing forces are integrated to accomplish pairing and alignment of homologous chromosomes.

### Ndj1 and Csm4 support homolog pairing through actin-directed motion

Rapid prophase movement of chromosomes is a prominent feature of mid-to-late meiotic prophase, yet little is known about the impact of chromosome motion during early meiotic prophase when chromosomes undergo pairing. We found that by eliminating one or all of three key components required for rapid prophase movement (Ndj1, Csm4 and actin polymerization) the kinetics of homolog pairing was delayed, consistent with findings using FISH and one-spot/two-spot TetR-GFP assays [64], [70]. In addition, we found that wild type and *ndj1*Δ cells gave indistinguishable pairing levels in the presence of the actin polymerization inhibitor Lat B, suggesting that the contribution of Ndj1 to pairing occurs entirely through its role in actin-directed chromosome movement. Conversely, we found that Lat B caused a more severe pairing delay in *ndj1*Δ and *csm4*Δ mutants compared to untreated cells, also suggesting that actin may play roles in chromosome pairing independent of Ndj1 and Csm4 (see below).

We suggest that actin-independent motion (perhaps diffusion or changes in chromatin compaction) is sufficient for allowing chromosome pairing, but that the process is accelerated when chromosomes are actively moving. In *C. elegans*, where pairing does not rely on DSB repair, homolog pairing appears to be driven by a combination of dynein-driven motion and diffusion that initiates at pairing centers [97]; while the loss of active pairing center motion leads to pairing delays, diffusion-based motion is sufficient for pairing [97], [98]. In *S. pombe*, mutations that disrupt dynein-driven nuclear movement of chromosomes also decrease the efficiency of the pairing process [20], [99]–[101]. We suggest an analogous situation occurs in budding yeast except that actin-mediated forces are involved. Some recent studies have drawn similar conclusions [102], [103].

### Actin can antagonize chromosome interactions

While the outcomes of our one-spot/two-spot TetR-GFP visual assay indicate a positive role for actin in pairing independent of Ndj1/Csm4, outcomes from the collision assay indicated that actin might also prevent or destabilize nonspecific interactions. That is, in the absence of Ndj1 and/or Csm4, we observed that Lat B increased allelic collisions yet slowed the process of pairing. One way to reconcile these two observations is that spurious or nonproductive interactions may be destabilized by actin-mediated mechanism not related to Ndj1/Csm4-dependent motion. While the nature of this mechanism is not clear, one possibility is that interstitial chromosomal sites are subject to motion via the actin cables that surround the nucleus using a KASH protein complex other than Csm4 (and Ndj1) [62]. Alternatively, interaction between interstitial chromosome sites might be prevented by sequestering them in different nuclear compartments and/or the nuclear envelope by association with an actin-associated structure or nuclear localized actin [104]. In interphase cells of yeast and *Drosophila*, diffusive motion of chromosomes is constrained to a limited subregion of the nucleus and treatment with the microtubule depolymerizing agent nocodazole alleviates this confinement [105]. Perhaps, during yeast meiosis an actin-mediated process acts similarly to constrain interstitial chromosome loci to enhance pairing. Additionally, several nuclear processes including chromatin remodeling have been shown to require actin and/or sensitivity to Lat B [106]–[108]. Chromatin remodeling may be necessary for stable pairing of loci but may not be essential for their collision.

### Multiple opposing forces promote strong interactions and eliminate weak interactions

**Figure 7:**
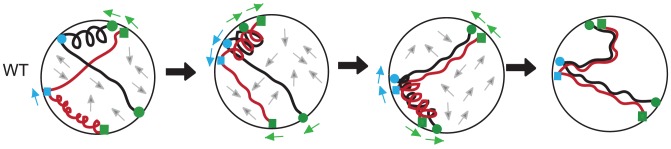
A mechanical model for homolog pairing. A hypothetical sequence of interactions between homologous chromosomes (shown as black or red) subjected to a coupled-spring oscillator (see text). In the sequence from left to right, homolog pairing becomes progressively stabilized as weak interactions are disrupted. The positive and negative forces of actin influence both homologs, with actin-based associations shown at telomeres (green; squares for the red chromosome and circles for the black homologous chromosome) and at interstitial sites (blue; squares for the red chromosome and circles for the black homologous chromosome). Arrows around the periphery of the nucleus indicate direction of movement for the telomeres (green) and the interstitial sites (blue). Grey arrows in the interior of the nucleus show “Brownian-like” motion/unknown forces on the chromosomes [105], [119]. (i) In wild-type cells, segments of chromosomes that are in closer proximity have axial segments that are more compact. (ii) Compact segments of homologous chromosomes interact. (iii) Movement of chromosome attachment points on the nuclear envelope results in stretching of segments that remove unstable interactions between chromosomes. (iv) Stable interactions between allelic loci are those achieved up to the point of dHJ resolution as established using the Cre/*loxP* assay [21].

We can envision a scenario where interstitial chromosome sites are coupled to one or more actin-based assemblies and their adjacent telomeres are attached to cytoplasmic actin cables via the Ndj1/Mps3/Csm4 protein bridge (Figure 7; Figure S4). The two systems acting simultaneously could direct discordant movement between chromosomes such that strong interactions persist while weak interactions are taken apart (Figure 7). Over time, chromosomes would be subject to alternating scrunching and stretching, perhaps analogous to a coupled-spring oscillator. Initially, chromosomes might undergo oscillations independent of one another, increasing the likelihood of productive strand invasion events to promote close, stable homolog juxtaposition. When pairing has been mostly achieved by zygotene [59], rapid prophase movement could serve to remove chromosome interlocks [60].

### Evidence for a Rec8-dependent spring component in regulating chromosome interactions

One of the most surprising findings in our study was the high level of allelic and ectopic chromosome interactions observed in the absence of Rec8, even in a *spo11*Δ background. To better understand this result, mechanistic insight may be gained by considering the function of “SMCs” (cohesins and condensins) in distributing spindle-pulling forces across the pericentromeric chromatin loops during mitosis [109]. Bloom and colleagues describe SMCs as having the physical attributes of “slip rings” (molecular pulleys) that impart the distribution of tension and regulate elasticity of these pericentromeric loops [110]. Analogously, Rec8 may distribute tension along or within the loop-axis structure of meiotic chromosomes as they are pulled by actin-driven motors, or even subjected to thermal motion (Figure S4). In the absence of Rec8, transduction of actin-mediated forces along chromosome segments would be diminished, as would their spring-like properties required for promoting allelic and taking apart ectopic interactions. Indeed, *rec8*Δ mutants in *S. pombe* exhibit defects in both chromosome compaction and pairing [111], [112]. Our observation that addition of Lat B increases nonspecific interactions in *rec8*Δ, and even more so in a *rec8*Δ *ndj1*Δ double mutant, suggests independent contributions of actin, cohesin and telomere function in promoting and limiting chromosome interactions during meiosis.

## Materials and Methods

### Yeast strains

All yeast strains are isogenic derivatives of SK1 (Table S3) [113]. Parental haploid strains SBY1338 (*MAT a ho::hisG lys2 ura3*Δ*::hisG leu2::hisG ade2*Δ*::hisG trp1::hisG GAL3 flo8::LEU2-loxP-ura3 ndt80*Δ*::LEU2-loxP-ade2*) and SBY1448 (*MAT alpha ho::hisG lys2::GAL1-Cre-LYS2 ura3*Δ*::hisG leu2::hisG ade2*Δ*:hisG leu2::hisG ade2re-LYS2 ura3-loxP-ura3 ndt80*Δ*::LEU2*) were used for transformation to generate PCR-mediated knockouts [21]. Knockout mutations in SBY1338 and SBY1438 were generated by transformation using PCR-based disruption that replaced the entire open reading frame with the *kanMX4*, *natMX*, or *hphMX4* marker [114], [115]. Integration of the drug-resistant markers into the appropriate genomic location and loss of wild-type markers were confirmed by PCR for every knockout strain created. The *cdc6-mn* (meiotic null) was generated by replacing the endogenous promoter of *CDC6* with the promoter of *SCC1*, which is down regulated during meiosis. The *sgs1-mn* allele was generated by placing *SGS1* under the control of the *CLB2* promoter [116]. The *rec8*Δ*::pREC8-SCC1* construct allows for expression of Scc1 instead of Rec8 by placing *SCC1* under the control of the Rec8 promoter [84].

Diploid strains carry an allelic pair of *loxP* sites on chromosome V (replacing *FLO8*; coordinates 377614 to 375215) and an ectopic *loxP* site on chromosome VIII (replacing *NDT80*; coordinates 356561 to 358444). Both chromosomes are ∼580 kb in length with centromeres located at ∼110 and ∼150 kb from the right telomere, respectively. The *loxP* sites are integrated in the left arm at sites roughly equidistant from their adjacent centromeres (*CEN5-FLO8* is ∼230 kb; *CEN8-NDT80* is ∼250 kb) and their adjacent telomeres (∼200 kb for both intervals). In both cases, the sites are located in a region of the genome that is unremarkable for DSB distribution [85], [87].

### Meiotic time courses and media

Media preparation and meiotic cell culture synchronization was performed as previously described [117]. Galactose was added to final concentration of 0.03% at one hour after transfer to sporulation media (SPM) to induce expression of Cre-recombinase. At *t* = 2 hrs. after transfer to SPM, cells were either treated with 0.1% DMSO or 30 uM Latrunculin B dissolved in DMSO.

### Quantitative PCR analysis

Genomic DNA for qPCR standard curves was isolated from haploids SBY 2576 (*ho::hisG lys2-pGAL1-Cre-LYS2 ura3*Δ*::hisG leu2::hisG ade2*Δ*::hisG trp1::hisG GAL3 flo8::LEU2-pGPD1-ura3*) for the allelic Cre/*LoxP* recombinant and SBY 2575 (*ho::hisG lys2-pGAL1-Cre-LYS2 ura3*Δ*::hisG leu2::hisG ade2*Δ*::hisG trp1::hisG GAL3 flo8::LEU2-pGPD1-loxP-ade2*) for the ectopic Cre/*loxP* recombinant. Cells were harvested 10 hours after transfer to SPM (t = 10 hrs.) for DNA extraction (unless otherwise noted). DNA purification was performed by vortexing cells in the presence of 0.5 mm zirconia/silica beads (BioSpec Products, Inc.) and phenol/chloroform, followed by ethanol precipitation of the DNA. DNA from haploid strains containing only the recombinant was serially diluted to make standard curves for the corresponding primer set and *ACT1*. The following are the sequence of the primer used:

Allelic and Ectopic Forward primer: 5′-CCAAGAACTTAGTTTCGACGGATC-3″

Allelic Reverse primer: 5′-TCGACATGATTTATCTTCGTTTCC-3′

Ectopic Reverse primer: 5′-CAATTGTCCCCCTCCTAATATACCA-3′

*ACT1* Forward primer: 5-AATGCAAACCGCTGCTCAAT-3′

*ACT1* Reverse primer: 5′-CAAAGCTTCTGGGGCTCTGA-3′

Primers for *ACT1* reaction are used at 100 nM concentration. Primers for detection of the ectopic recombinant are used at 500 nM concentration. For detection of the allelic recombinant, the forward primer was used at 500 nM and the reverse primer was used at 900 nM. Quantitative PCR was performed on the ABI 7300 using SYBR Green Power master mix (ABI). The cycling conditions are as follows: 95° for 10 min. Followed by 40 cycles of 95° 15 sec and 60° 1 min. Collisions in all strains except for strains containing the *cdc-6mn* mutation were calculated as 4 times the recombinant copy number divided by the copy number of *ACT1* to yield number of recombinants per four chromatids. Due to the absence of meiotic replication in *cdc-6mn* mutants, collisions in *cdc-6mn* mutants were calculated as 2 times the recombinant copy number divided by the copy number of *ACT1* to yield number of recombinants for the two chromatids of the unreplicated homolog pair.

### Visual homolog pairing assay

Cells were synchronized and Lat B was added as described above except that galactose was not added to the cultures. Cells were removed (250 ul) every hour, fixed in 4% paraformaldehyde (PFA) in phosphate buffered saline (PBS; pH 7.4) for 8 minutes at room temperature. Cells were then washed once with PBS and stored at 4°C until they could be analyzed. Cell morphology and pairing levels were the same in unfixed and in fixed cells for up to one week (data not shown). A monolayer of cells on a slide was prepared according to [118] and cells were immediately imaged at 100× magnification using a hybrid spinning disk confocal microscope (Intelligent Imaging Innovations) with a 488 nm laser for 150 msec exposure time per slice. Pairing was assessed visually in projected Z-stacks by determining the fraction of cells containing one GFP spot. Each Z-stack consisted of ∼30 slices with 0.25 µm separating each slice. Strains used for visualizing homolog pairing were:

SBY4503×SBY4504 (*MAT a/MAT alpha ho::hisG/" LEU2::tetR-GFP/" URA3::tetOx224/" his3::hisG/" ndt80*Δ*::NAT/"*)

SBY4506×SBY4507 (*MAT a/MAT alpha ho::hisG/" LEU2::tetR-GFP/" URA3::tetOx224/" his3::hisG/" ndt80*Δ*::NAT/" ndj1*Δ*::Hph/"*)

SBY4870×SBY4871 (*MAT a/MAT alpha ho::hisG/" LEU2::tetR-GFP/" URA3::tetOx224/" his3::hisG/" ndt80*Δ*::NAT/" csm4*Δ*::Hph/"*)

SBY4872×SBY4873 (*MAT a/MAT alpha ho::hisG/" LEU2::tetR-GFP/" URA3::tetOx224/" his3::hisG/" ndt80D::NAT/" ndj1*Δ*::Hph/" csm4*Δ*::Hph/"*).

### Return to growth analysis

Cells were harvested 10 hours after transfer to SPM (unless otherwise noted). Cell aliquots were pelleted, resuspended in 2% glucose, sonicated 5 seconds at 15% maximum power using the microtip of a 550 Sonic ZD-dismembrator (Fisher Scientific), and diluted appropriately prior to plating on selective (SC-Ura) and nonselective media (YPD-Ade).

### Statistical analysis

A two-tailed Student's t-test was performed for determining the *P*-value between treated and untreated cultures. All bar plots signify the mean ± standard deviation of the mean for measured collision levels (above). The total number of independent cultures for all strains is listed in Table S2. Heatmaps indicating the P-values for comparison of values across multiple strains was obtained from applying a two-tailed Student's t-test followed by Sidak correction where *P = 1−(1−α)^1/n^*.

## Supporting Information

Figure S1 Allelic collision levels in various DSB repair mutants using the RTG and qPCR assays. RTG levels. A. Both allelic and ectopic collision levels are shown for wild type and *spo11*Δ strains using qPCR and the return-to-growth (RTG) method. Sidak-corrected P-values incorporating these strains in the study are shown in Figure S3. B. Allelic collision levels in strains where DSB repair is altered compared to wild type. Sidak-corrected P-values incorporating these strains in the study are shown in Figure S3. The number of replicas is reported in Table S3.
(EPS)

Figure S2 Allelic and Ectopic collision levels for all mutants. Graphical parameters are as described in Figure 3.
(EPS)

Figure S3 Heatmap of allelic and ectopic collision levels for all mutants. Heatmap of Sidak adjusted *P*-values from Student's t-test comparing collision levels between relevant mutants in untreated cells.
(EPS)

Figure S4 A mechanical model for homolog pairing in *ndj1*Δ and *csm4*Δ mutant backgrounds. Beginning (white panels) and ending (grey panels) snapshots of the pairing process in WT, *ndj1*Δ and *csm4*Δ mutant cells. Left pair of panels depicts untreated cells. Right pair of panels illustrates cells treated with Lat B. See Figure 7 for description of objects.
(EPS)

Table S1 Allelic and Ectopic collision levels with and without galactose induction of *Cre*. Analysis of collision levels in WT, *spo11*Δ, *spo11*Δ *ndj1*Δ, *spo11*Δ *zip1*Δ, and *spo11*Δ *ndj1*Δ *zip1*Δ strains with and without galactose induction of *Cre* at 2 hrs post meiotic induction.
(PDF)

Table S2 Allelic and ectopic collision levels all mutants analyzed. The n values denote the total numbers of independent cultures analyzed for each strain. For most strains up to three cultures per strain per day were analyzed in parallel on the same day. WT or *spo11*Δ strains, as appropriate, were included in every experiment as a control. Strains with an n value of less than 6 were analyzed in duplicate or singly on at least two different days, respectively. To normalize collision levels per meiosis in the absence of DNA replication in *cdc6-mn* strains, collision levels are determined as the measured recombinants per 2 chromatids, not 4 as is the case for all other reported values.
(EPS)

Table S3 Strains used for collision analysis.
(PDF)
