# Supplementary material for: Multiple Opposing Constraints Govern Chromosome Interactions during Meiosis
Source: PLoS Genet. 2013 Jan 17;9(1):e1003197. doi: 10.1371/journal.pgen.1003197 (PMC3547833; doi:10.1371/journal.pgen.1003197)
Supplement: Table S1 — Allelic and Ectopic collision levels with and without galactose induction of Cre. Analysis of collision levels in WT, spo11Δ, spo11Δ ndj1Δ, spo11Δ zip1Δ, and spo11Δ ndj1Δ zip1Δ strains with and without galactose induction of Cre at 2 hrs post meiotic induction. (PDF) [file pgen.1003197.s005.pdf]

## Supplemental Table 1

### Allelic Collisions

| genotype | n | without galactose | with galactose |
|---|---|---|---|
| *WT* | 3 | 0.00139 ± 0.00048 | 0.1398 ± 0.0093 |
| *spo11Δ* | 3 | 0.00071 ± 0.00021 | 0.0328 ± 0.0037 |
| *spo11Δ ndj1Δ* | 3 | 0.00034 ± 0.00012 | 0.0204 ± 0.0044 |
| *spo11Δ zip1Δ* | 3 | 0.00050 ± 0.00016 | 0.0236 ± 0.0045 |
| *spo11Δ ndj1Δ zip1Δ* | 3 | 0.00050 ± 0.00016 | 0.0218 ± 0.0026 |

### Ectopic Collisions

| genotype | n | without galactose | with galactose |
|---|---|---|---|
| *WT* | 3 | $6.14 \times 10^{-5} \pm 5.55 \times 10^{-5}$ | 0.0215 ± 0.00603 |
| *spo11Δ* | 3 | $5.24 \times 10^{-5} \pm 5.32 \times 10^{-5}$ | 0.0178 ± 0.00283 |
| *spo11Δ ndj1Δ* | 3 | 0 | 0.0124 ± 0.00164 |
| *spo11Δ zip1Δ* | 3 | 0 | 0.0151 ± 0.00094 |
| *spo11Δ ndj1Δ zip1Δ* | 3 | $4.46 \times 10^{-5} \pm 5.14 \times 10^{-5}$ | 0.0132 ± 0.00176 |
