# Supplementary material for: Multiple Opposing Constraints Govern Chromosome Interactions during Meiosis
Source: PLoS Genet. 2013 Jan 17;9(1):e1003197. doi: 10.1371/journal.pgen.1003197 (PMC3547833; doi:10.1371/journal.pgen.1003197)
Supplement: Table S3 — Strains used for collision analysis. (PDF) [file pgen.1003197.s007.pdf]

Supplemental Table 3

Strain	Genotype
SBY1476	*MAT a/α ho::hisG/ ho::hisG lys2::GAL1-Cre-LYS2/lys2 ura3Δ::hisG/ ura3Δ::hisG leu2::hisG/leu2::hisG ade2Δ::hisG/ade2Δ::hisG trp1::hisG/trp1::hisG GAL3/ GAL3 flo8::LEU2-pGPD1-loxP-lacZ/flo8::LEU2-loxP-ura3 ndt80Δ::LEU2-loxP-ade2/ ndt80Δ::LEU2*<br><br>Diploid from mating SBY1338 and SBY1438 (Peoples *et al.* 2002)
SBY1839	SBY1476 except that it has *rad54Δ::kanMX4/ rad54Δ::kanMX4*
SBY2267	SBY1476 except that it has *pTRP21*
SBY2268	SBY1476 except that it has *pTRP33*
SBY2660	SBY1476 except that it has *rad54Δ::HphMX4/ rad54Δ::HphMX4 rdh54Δ::kanMX4/ rdh54Δ::kanMX4*
SBY2857	*MAT a/α ho::hisG/ ho::hisG lys2::GAL1-Cre-LYS2/lys2 ura3Δ::hisG/ ura3Δ::hisG leu2::hisG/leu2::hisG ade2Δ::hisG/ade2Δ::hisG trp1::hisG/trp1::hisG GAL3/ GAL3 flo8::LEU2-pGPD1-loxP-lacZ/flo8::LEU2-loxP-ura3 ndt80Δ::LEU2/ ndt80Δ::LEU2*
SBY2858	*MAT a/α ho::hisG/ ho::hisG lys2::GAL1-Cre-LYS2/lys2 ura3Δ::hisG/ ura3Δ::hisG leu2::hisG/leu2::hisG ade2Δ::hisG/ade2Δ::hisG trp1::hisG/trp1::hisG GAL3/ GAL3 flo8::LEU2-pGPD1-loxP-lacZ/flo8::LEU2-loxP-ade2 ndt80Δ::LEU2/ ndt80Δ::LEU2*
SBY2880	*MAT a/α ho::hisG/ ho::hisG lys2::GAL1-Cre-LYS2/lys2 ura3Δ::hisG/ ura3Δ::hisG leu2::hisG/leu2::hisG ade2Δ::hisG/ade2Δ::hisG trp1::hisG/trp1::hisG GAL3/ GAL3 arg4Δ::LEU2-pGPD1-loxP-lacZ/ arg4Δ::LEU2-loxP-ade2 ndt80Δ::LEU2/ ndt80Δ::LEU2*
SBY3118	SBY1476 except that it has *cdc6Δ::kanMX6::pScc1::3HA-CDC6/ cdc6Δ::kanMX6::pScc1::3HA-CDC6*
SBY3129	SBY1476 except that it has *csm4Δ::hphMX4/csm4Δ::hphMX4*
SBY3293	SBY1476 except that it has *zip1Δ::kanMX4/zip1Δ::kanMX4 spo11Δ::hphMX4/spo11Δ::hphMX4*
SBY3342	SBY1476 except that it has *zip1Δ::kanMX4/zip1Δ:kanMX4 ndj1Δ::hphMX4/ndj1Δ::hphMX4 spo11Δ::natMX4/spo11Δ::natMX4*
SBY3388	SBY1476 except that it has *pCLB2-SGS1-kanMX/pCLB2-SGS1-kanMX*
SBY3393	SBY1476 except that it has *pCLB2-SGS1-kanMX/pCLB2-SGS1-kanMX zip1Δ::kanMX4/zip1Δ::kanMX4*
SBY3394	SBY1476 except that it has *spo11Δ::kanMX4/spo11Δ::kanMX4 rec8Δ::hphMX4/rec8Δ::hphMX4*
SBY3461	SBY1476 except that it has *red1Δ::kanMX4/red1Δ::kanMX4 spo11Δ::hphMX4/spo11Δ::hphMX4*

SBY3462	SBY1476 except that it has *hop1Δ::kanMX4/hop1Δ::kanMX4 spo11Δ::hphMX4/spo11Δ::hphMX4*
SBY3463	SBY1476 except that it has *spo11Δ::kanMX4/spo11Δ::kanMX4 csm4Δ::hphMX4/csm4Δ::hphMX4*
SBY3464	SBY1476 except that it has *ndj1Δ::kanMX4/ndj1Δ::kanMX4 csm4Δ::nat/csm4Δ::nat*
SBY3521	SBY1476 except that it has *ndj1Δ::kanMX4/ndj1Δ:kanMX4 rec8Δ::hphMX4/rec8Δ::hphMX4*
SBY3524	SBY1476 except that it has *ndj1Δ::kanMX4/ndj1Δ:kanMX4 spo11Δ::hphMX4/spo11Δ::hphMX4 rec8Δ::nat/rec8Δ::nat*
SBY3738	SBY1476 except that it has *rec8Δ::hphMX4/rec8Δ::hphMX4 cdc6Δ::kanMX6::pScc1::3HA-CDC6/cdc6Δ::kanMX6::pScc1::3HA-CDC6*
SBY3744	SBY1476 except that it has *spo11Δ::nat/spo11Δ::nat cdc6Δ::kanMX6::pScc1::3HA-CDC6/cdc6Δ::kanMX6::pScc1::3HA-CDC6*
SBY3745	SBY1476 except that it has *spo11Δ::nat/spo11Δ::nat rec8Δ::hphMX4/rec8Δ:hphMX4 cdc6Δ::kanMX6::pScc1::3HA-CDC6/cdc6Δ::kanMX6::pScc1::3HA-CDC6*
SBY1461	SBY1476 except that it has *spo11Δ::kanMX6/spo11Δ::kanMX6*
SBY1489	SBY1476 except that it has *ndj1Δ::kanMX6/ndj1Δ::kanMX6*
SBY1488	SBY1476 except that it has *zip1Δ::kanMX6/zip1Δ::kanMX6*
SBY1735	SBY1476 except that it has *zip3Δ::kanMX6/zip3Δ::kanMX6*
SBY1653	SBY1476 except that it has *rdh54Δ::kanMX6/rdh54::kanMX6*
SBY2043	SBY1476 except that it has *zip1Δ::kanMX6/zip1Δ::kanMX6 ndj1Δ::hphMX4/ndj1Δ::hphMX4*
SBY2069	SBY1476 except that it has *ndj1Δ::kanMX6/ndj1Δ::kanMX6 spo11Δ::hphMX4/spo11Δ::hphMX4*
SBY3392	SBY1476 except that it has *rdh54Δ::kanMX6/rdh54Δ::kanMX6 spo11Δ::hphMX4/spo11Δ::hphMX4*
SBY3465	SBY1476 except that it has *ndj1Δ::kanMX6/ndj1Δ::kanMX6 spo11Δ::hphMX4/spo11Δ::hphMX4 csm4Δ::nat/csm4Δ::nat*
SBY2922	SBY1476 except that it has *rec8Δ::hphMX4/rec8Δ::hphMX4*
SBY3518	SBY1476 except that it has *mek1Δ::kanMX6/mek1Δ::kanMX6 spo11Δ::nat/spo11Δ::nat*
SBY1559	SBY1476 except that it has *rad52Δ::kanMX6/rad52Δ::kanMX6*
SBY1799	SBY1476 except that it has *sae2Δ::kanMX6/sae2Δ::kanMX6*
SBY1513	SBY1476 except that it has *rad51Δ::kanMX6/rad51Δ::kanMX6*
SBY1715	SBY1476 except that it has *rad55Δ::kanMX6/rad55Δ::kanMX6*
SBY3990	SBY1476 except that it has *pREC8-SCC1-3HA::LEU2/pREC8-SCC1-3HA::LEU2 rec8Δ::kanMX6/rec8Δ::kanMX6*

SBY3992	SBY1476 except that it has *rec8Δ::hphMX4/rec8Δ::hphMX4 dmc1Δ::kanMX/dmc1Δ::kanMX*
SBY1487	SBY1476 except that it has *dmc1Δ::kanMX/dmc1Δ::kanMX*
SBY4344	SBY1476 except that it has *pREC8-SCC1-3HA::LEU2/pREC8-SCC1-3HA::LEU2 rec8Δ::kanMX6/rec8Δ::kanMX6 spo11Δ::hphMX4/spo11Δ::hphMX4*
